# Distorter Characterisation Using Mutual Inductance in Electromagnetic Tracking

**DOI:** 10.3390/s18093059

**Published:** 2018-09-12

**Authors:** Herman Alexander Jaeger, Pádraig Cantillon-Murphy

**Affiliations:** 1 Department of Electrical and Electronic Engineering, School of Engineering, University College Cork, Cork, Ireland; p.cantillonmurphy@ucc.ie; 2 Tyndall National Institute, Dyke Parade, Cork, Ireland

**Keywords:** electromagnetic tracking, magnetics, distortion, robotics, image-guided interventions

## Abstract

Electromagnetic tracking (EMT) is playing an increasingly important role in surgical navigation, medical robotics and virtual reality development as a positional and orientation reference. Though EMT is not restricted by line-of-sight requirements, measurement errors caused by magnetic distortions in the environment remain the technology’s principal shortcoming. The characterisation, reduction and compensation of these errors is a broadly researched topic, with many developed techniques relying on auxiliary tracking hardware including redundant sensor arrays, optical and inertial tracking systems. This paper describes a novel method of detecting static magnetic distortions using only the magnetic field transmitting array. An existing transmitter design is modified to enable simultaneous transmission and reception of the generated magnetic field. A mutual inductance model is developed for this transmitter design in which deviations from control measurements indicate the location, magnitude and material of the field distorter to an approximate degree. While not directly compensating for errors, this work enables users of EMT systems to optimise placement of the magnetic transmitter by characterising a distorter’s effect within the tracking volume without the use of additional hardware. The discrimination capabilities of this method may also allow researchers to apply material-specific compensation techniques to minimise position error in the clinical setting.

## 1. Introduction

Electromagnetic tracking (EMT) is becoming a central platform technology for image-guided procedures and interventions in the modern clinical setting [1]. The technology enables precise navigation deep within the human body by providing real-time position and orientation data of tracked instruments used by the clinician. When used in combination with patient imaging, the technology enables navigation within the human body in areas traditionally beyond the reach of cameras. Tracked instruments are manufactured by integrating a magnetic sensor in a fixed position within the instrument body. Electromagnetic navigation bronchoscopy (ENB) is the largest clinical application arising from EMT technology [2,3], although applications such as neurosurgery [4] and ear-nose-and-throat [5] are seeing increased uptake. Outside of medical use, EMT is commonly used as a reference device in the development of robotic systems and manipulators. The absence of a camera provides vastly greater flexibility during experimental data acquisition, where the problem of visual obstruction due to robotic limbs is eliminated.

EMT operates on the principle of magnetic induction; a conductive loop (sensor) will experience an induced voltage when placed in a time-varying magnetic field according to Faraday’s law in Equation (1). The position of the sensor is directly related to this induced voltage signal. If the properties of the time-varying magnetic field are precisely known then it is possible to accurately calculate the position of the sensor based solely on this signal.

(1) $$v\left(t\right)=-\frac{d\Phi }{dt}$$

**Figure 1:**
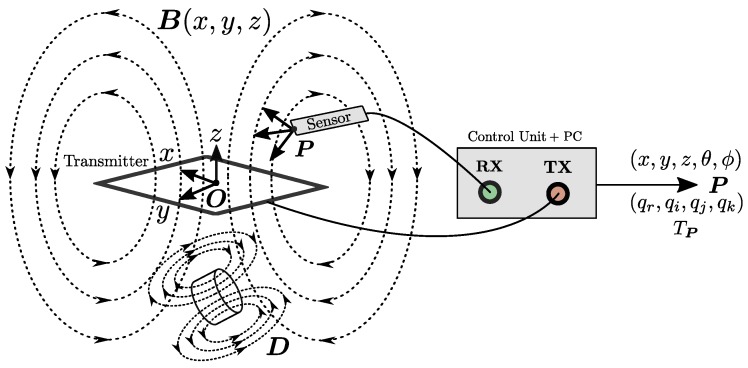
Generalised operation of an EM tracking system. A control unit is connected to a transmitter board (TX) and magnetic sensor (RX). The sensor experiences an induced voltage from the transmitter’s time-varying magnetic field B. The control unit measures this voltage and resolves the sensor position based on this measurement to yield the position and orientation of the sensor P with respect the origin O. The orientation component takes the form of a vector, quaternion or transformation. Distorters D in the region of the TX board distort the magnetic field and result in sensor positioning errors.

The first magnetic tracking system was developed by *Polhemus Navigation Systems* [6] which standardised the tracking system topology. An overview of how a typical EMT system operates is shown in Figure 1.

All magnetic navigation systems experience error due to distorters in the operating environment [7,8]. Distorters typically take the form of metallic objects which can be either static or moving with respect to the tracking system transmitter. Tables and surgical instruments make up the majority of distorters in the clinical setting as they are typically made from either aluminium or stainless steel due to sterility requirements. These metallic structures, when placed in the vicinity of the tracking system transmitter, may affect the spatial distribution of the transmitted magnetic field which can cause significant errors in the reported sensor positions [8,9]. Ferromagnetic distorters (those containing iron) change the shape of the local magnetic field distribution due to their relatively high magnetic permeability. Highly conductive non-ferromagnetic distorters such as aluminium produce opposing magnetic fields by induced eddy-currents. Commercial systems such as the Aurora system (Northern Digital Inc., Waterloo, ON, Canada), trakSTAR (Ascension Technologies, Shelburne, VT, USA) and Fastrak (Polhemus, Colchester, VT, USA) have been subject to standardised assessments to determine the effects of different distorter types [10,11]. Some of these tracking systems contain compensation techniques, although these methods remain proprietary and do not provide any localisation of the distorter location.

Compensation for the effects of metallic distorters has become a popular topic among researchers working on integrating EMT into the clinical setting. Intrinsic compensation techniques include manipulation of the underlying magnetics through the use of magnetic shielding [12] and novel field modulation methods [13,14] which act to nullify the effect of local metallic objects. Other techniques rely on the use of redundant position measurements in order to characterise and map the nature of the distortion while simultaneously compensating for positioning error: EMT-only techniques utilise multiple magnetic sensors in fixed relative orientation combination with simultaneous localisation and mapping (SLAM) to effectively reduce error by as much as 67% [15]. Optical tracking systems have also been used in combination with EMT to provide a position reference for sensor fusion algorithms [16].

While many of these compensation techniques are effective in reducing error from distorters, characterisation of the distortions themselves typically requires that an undistorted sample of tracking system positions be obtained and analysed in order to determine the degree of distortion present. This provides distortion information purely from the perspective of the magnetic sensor and not from that of the transmitter. It is also a relatively time-consuming process as a cloud of points needs to be acquired with respect to either a relative [15] or optical ground truth [16]. Procedural tables, trolleys and other medical equipment are the biggest distortion sources in the clinical setting, and their proximity to the transmitter board has a large impact on overall system accuracy [12]. This paper proposes a novel distortion detection scheme which utilises only the EMT transmitter. The design is based on an existing transmitter design for the open-source Anser EMT tracking system [17,18]. The modified design allows calculation of distortion error from the perspective of the transmitter based on measurements of the mutual inductance between constituent transmitter coils. Amplitude and phase measurements from the resulting signal waveforms allow discrimination between different materials. The calculations can be performed autonomously allowing EM tracking systems to detect the presence, material type and approximate location of distortions without the need for position acquisition or undistorted control data.

While this work does not attempt to compensate for position errors, the proposed technique can potentially be used in tandem with existing compensation methods. The proposed method can allow EMT users to quickly optimise placement of EMT transmitters such that environmental errors can be minimised prior to the application of further compensation techniques.

## 2. Background

### 2.1. Magnetic Transmitter

**Figure 2:**
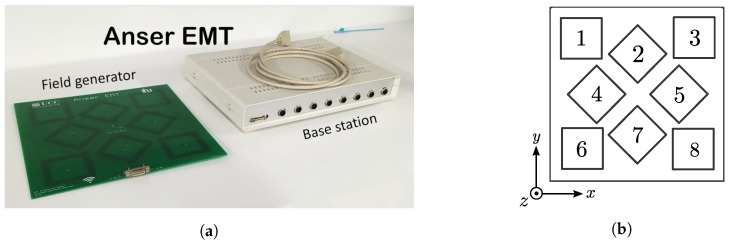
(**a**) The Anser electromagnetic tracking system [17]. (**b**) The arrangement of coils on the Anser EMT transmitter. This arrangement was originally based on an optimised coil placement by [20].

All EMT systems use a magnetic transmitter to produce a time-varying magnetic field as shown in Figure 1. The transmitting field must be spatially unique such that each position in space has a unique magnetic signature. Most system transmitter designs employ multiple transmitting coils with each generating a magnetic field at a different frequency to fulfil this requirement. EMT transmitters come in a number of different arrangements including tri-axial [6], planar coil [19] and planar printed circuit board (PCB) [17,18] such as shown in Figure 2a. In this paper we alter the role of the magnetic transmitter design such that each transmitter coil can also behave as a receiver. The theory of how this operates is described in the next section.

### 2.2. Mutual Inductance

**Figure 3:**
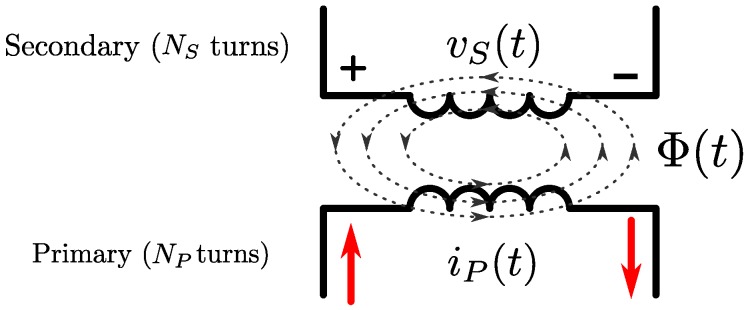
The interaction between two coupled inductors. A time-varying current $i_{P}\left(t\right)$ in the primary coil establishes a time-varying magnetic flux Φ(t), a portion of which links the secondary winding and causes a time-varying voltage to be induced at its terminals.

Mutual inductance is the electrical property which relates how an electrical current in a conductor influences the voltage on another conductor. Consider the coil arrangement shown in Figure 3. The alternating current $i_{P}\left(t\right)$ flowing through the primary coil creates a time-varying magnetic field. The resulting magnetic flux Φ(t) interacts with a secondary coil in the vicinity and causes a voltage $v_{S}\left(t\right)$ to be induced on it. The extent of this interaction is captured by the mutual inductance coefficient *M* in Equation (2) and is the basic principle of an electrical transformer [21].

(2) $$v_{S}\left(t\right)=N_{S}\frac{d\Phi (t)}{dt}=M\frac{di_{P}\left(t\right)}{dt}$$

In the case of a EMT transmitter containing multiple coils, the mutual inductances between all coils can be represented as a matrix M shown in Equation (3). The diagonal entries represent the self-inductances of each coil. The induced voltages on a particular coil can then be expressed as a superposition of its self-induced voltage and those induced by every coil in the transmitter according to Equation (4). Matrix representations of inductances are a commonly used tool in circuit analysis as they compactly define the interaction between all current-carrying structures within a device, from the small scales of integrated circuit packages [22] to high powered electrical machine windings [23].

(3) $$M=\left[\begin{array}{cccc} L_{1} & M_{12} & \ldots & M_{1N}\\ M_{21} & L_{2} & \ldots & M_{2N}\\ \vdots & \vdots & \ddots & \vdots \\ M_{N1} & M_{N2} & \ldots & L_{N} \end{array}\right]$$

(4) $$V=\left[\begin{array}{c} v_{1}\\ v_{2}\\ \vdots \\ v_{n} \end{array}\right]=\left[\begin{array}{cccc} L_{1} & M_{12} & \ldots & M_{1N}\\ M_{21} & L_{2} & \ldots & M_{2N}\\ \vdots & \vdots & \ddots & \vdots \\ M_{N1} & M_{N2} & \ldots & L_{N} \end{array}\right]\left[\begin{array}{c} di_{1}/dt\\ di_{2}/dt\\ \vdots \\ di_{N}/dt \end{array}\right]=M\frac{dI}{dt}$$

Ideally the induction matrix is symmetric about the diagonal since the definition of mutual inductance implies that $M_{\alpha \beta }=M_{\beta \alpha }$. If an EMT magnetic transmitter is capable of switching its coils between transmitter (TX) and receiver (RX) modes of operation, then the mutual inductance between every coil can be experimentally recovered in matrix form.

### 2.3. Transmitter Inductance Matrix

The mutual inductance matrix defines how effectively the magnetic flux is coupled from one circuit to another and is therefore dependent on the intrinsic parameters of the circuits being analysed. The Anser EMT system transmitter shown in Figure 2 uses eight identical transmitter coil circuits in a well-known optimised arrangement [20]. According to Equation (2), obtaining the mutual inductance between two coils requires applying a time-varying current to the primary coil while simultaneously measuring the induced voltage on the secondary coil. For a tracking system containing *N* coils there must be N!/(N−2)! mutual inductance measurements acquired, since two coils are utilised for a single inductance measurement. Generalising, the voltage induced on coil α due to a transmitting coil β is given by Equation (5). This relation can be represented in matrix from in Equation (6).

(5) $$v_{\alpha \beta }\left(t\right)=M_{\alpha \beta }\frac{di_{\beta }\left(t\right)}{dt}$$

(6) $$\left[\begin{array}{cccc} 0 & v_{12} & \ldots & v_{1N}\\ v_{21} & 0 & \ldots & v_{2N}\\ \vdots & \vdots & \ddots & \vdots \\ v_{N1} & v_{N2} & \ldots & 0 \end{array}\right]=\left[\begin{array}{cccc} 0 & M_{12} & \ldots & M_{1N}\\ M_{21} & 0 & \ldots & M_{2N}\\ \vdots & \vdots & \ddots & \vdots \\ M_{N1} & M_{N2} & \ldots & 0 \end{array}\right]\left[\begin{array}{cccc} di_{1}/dt & 0 & 0 & 0\\ 0 & di_{2}/dt & \ldots & 0\\ \vdots & \vdots & \ddots & \vdots \\ 0 & 0 & \ldots & di_{N}/dt \end{array}\right]$$

If both the source current and induced voltage can be measured, then the mutual inductance matrix can then be calculated using simple matrix inversion in Equation (7):

(7) $$M={\left[\begin{array}{ccc} \backslash & & \\ & \frac{di_{j}}{dt} & \\ & & \backslash \end{array}\right]}^{-1}V$$

### 2.4. Summary

The eight transmitter coils of the Anser tracking system result in a total of 56 mutual inductance calculations. Metallic objects in the vicinity of the transmitter will change the measured mutual inductance due to ferromagnetic and non-ferromagnetic (eddy-current) effects, as they act to distort shape and strength of the surrounding magnetic field. Detecting these changes in inductance is a commonly found design in very-low-frequency metal detecting circuits [24,25]. Measuring this change in inductive coupling is the foundation of this work. Initially we simulate the inductive coupling between transmitter coils from first principles. The experimental hardware is then discussed in detail followed by a thorough description of the experimental procedures performed. Finally the measured inductance error results are correlated with position errors experienced by the tracking system.

## 3. Transmitter Inductance Simulation

**Figure 4:**
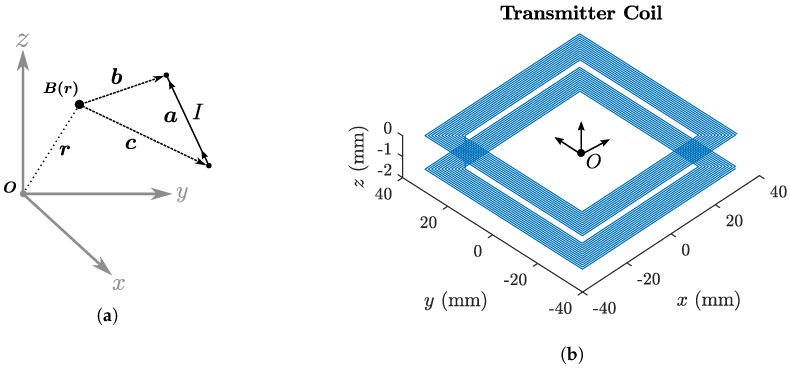
(**a**) Vector diagram showing the vectors in Equation (8). a is a vector representing the length and direction of a current carrying conductive filament. r is the vector from the origin to the observation point from which the field due to the filament is measured. Vectors c and b point from the observer position r to the start and end points of the current filament respectively. (**b**) 3D model of the double-sided PCB traces of a single transmitter coil with the origin *O* located at its centre. Each straight-line trace can be considered a single current filament.

The mutual coupling between each coil of the transmitting field generator shown in Figure 2 was simulated to investigate whether detection of mutually induced voltages was feasible and to provide a reference for experimental measurements. For simulation, each transmitter coil of the tracking system can be considered as a set of individual straight copper traces, with each trace idealised as a current-carrying filament [26]. The magnetic field (in Tesla) observed at a point r due to a single current-carrying filament is given by Equation (8), where *I* is the magnitude of the filament current, $\mu_{0}$ is the magnetic permeability of free space and a, b and c are vectors relating the location of the current filament to the observation point r as shown in Figure 4a. The magnetic field due to each PCB coil can then be modelled as a superposition of fields produced by all constituent current filaments [26,27] (An implementation of this equation can be found online: https://osf.io/47q8q/).

(8) $$B\left(r\right)=\frac{\mu_{0}I}{4\pi }\left(\frac{c\times a}{{\left|c\times a\right|}^{2}}\right)\left(\frac{a\cdot c}{\left|c\right|}-\frac{a\cdot b}{\left|b\right|}\right)$$

The magnetic coupling between any two coils can be simulated by integrating the magnetic field of the primary coil over the area of the secondary coil. Since all transmitting coils are co-planar in the *x*-*y* plane, the total flux produced by a primary coil *P* intersecting a secondary coil *S* can be written as an area integral in Equation (9), where $B_{P}$ is the primary’s magnetic field strength at location (x,y) and $dA_{S}$ is a differential area element of the secondary coil.

(9) $$\Phi_{S}=\int_{A_{S}}B_{P}\left(x,y\right)\cdot dA_{S}$$

The equation for the magnetic field is highly non-linear and must be numerically integrated as discrete a sum over the length *l* and width *w* of the secondary coil. The total flux linkage $\lambda_{S}$ is computed by scaling the result by the number of secondary coil turns *N* as shown in Equation (10).

(10) $$\lambda_{S}=N_{S}\Phi_{S}≃N_{S}\sum_{l}\sum_{w}B_{P}\left(x,y\right)\cdot \Delta x\Delta y$$

**Figure 5:**
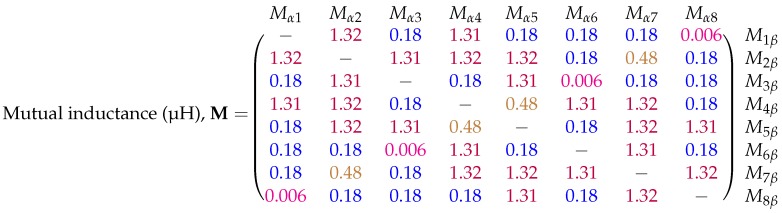
Simulated mutual inductance matrix for the eight coil PCB transmitter of the Anser EMT system. The entries have units of microhenries (μH). Self-inductances of each coil are omitted. Precision of entries have been truncated with similar values colour-coded for clarity. Each coil area was subdivided into 4 million area elements for the integration process.

**Figure 6:**
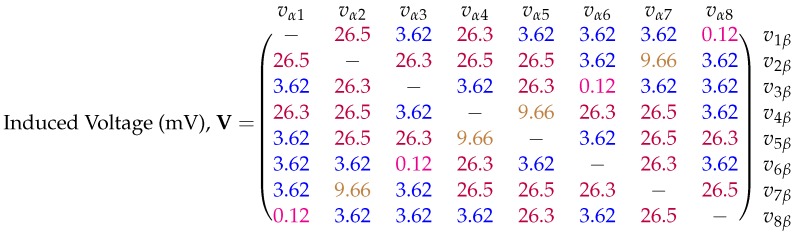
Simulated induced peak-voltage matrix for the eight coil PCB transmitter of the Anser EMT system based on the results in Figure 5. Precision of entries have been truncated with similar values colour-coded for clarity. The entries have units of millivolts (mV).

In this work, numerical computation of the integrals was performed using Matlab (MathWorks, Natick, MA, USA). Consistent with the experimental design described previously [18], the coil integration area was approximated as 25 uniform turns of the mean coil side length of 66.75 mm. Full coil dimensions are shown in Table 1. Acceleration of the calculations was performed using the *parfor* language feature of the Parallel Computing toolbox in Matlab. Through rearrangement of Equation (2) the mutual inductance between two coils α and β be calculated in Equation (11).

(11) $$M_{\alpha \beta }=\frac{N_{\alpha }\Phi_{\alpha }}{I_{\beta }}$$

where $N_{\alpha }$ and $\Phi_{\alpha }$ are the turn count and magnetic flux of the secondary coil and $I_{\beta }$ is the current flowing in the primary coil. The resulting mutual inductance matrix shown in Figure 5. The colour-coding of the entries reveals that there are only four observed unique mutual inductance values for the transmitter (ignoring the 10 nH difference between 1.31 μH and 1.32 μH entries, which is attributed to modelling error). These unique entries are a consequence of the square symmetry in the transmitter design. Referring to Figure 2b it can be expected that adjacent coils will experience the highest mutual inductance values, while the furthest separated coils will experience the lowest values. For example, coils 1 and 2 have a mutual inductance, indicated by $M_{12}$, of 1.32 μH, while coils 1 and 8 have a comparatively low mutual inductance $M_{18}$ of 6 nH. Taking each entry $M_{\alpha \beta }$ one can calculate the predicted induced voltage due to a sinusoidal time-varying current i(t). Assuming a peak coil current *I* of 160 mA [18] at a frequency of 20 kHz, Equation (12) provides the induced peak voltage for a given mutual inductance $M_{\alpha \beta }$:

(12) $$v_{\alpha \beta }=M_{\alpha \beta }\frac{di_{\beta }}{dt}=\omega IM_{\alpha \beta }$$

where ω=2πf, the angular frequency of the primary coil current. Figure 6 shows the matrix of predicted voltages for the different inductances shown in Figure 5. On the order of millivolts, these induced voltages are well within acceptable sensing ranges enabling reliable measurements to be taken. The measurement hardware used to experimentally measure these voltages is discussed next.

## 4. Experimental Setup

### 4.1. Modified Transmitter Design

The tracking system field generator consists of a planar transmitter coil array fabricated on a single PCB as shown in Figure 2. The high process accuracy ensures experimental repeatability and similar self-inductance values on each coil winding. The properties of each coil are shown in Table 1. This transmitter design was modified with additional circuitry to enable the transmitter’s mutual inductance to be experimentally calculated. Each coil circuit was modified to allow independent switching between modes of transmission (TX) and reception (RX). By selectively controlling each coil in this manner the mutual inductances of the transmitter can be experimentally acquired.

**Figure 7:**
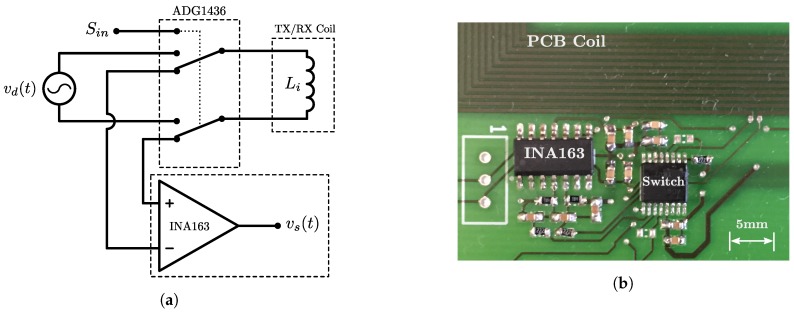
(**a**) Simplified circuit diagram of the coil switching circuit. The ADG1436 behaves as a double-pole double throw (DPDT) switch enabling the coil to switch between TX and RX modes. $S_{in}$ is the logic signal used to control the switch position. In TX mode, the switch connects the driving source $v_{d}\left(t\right)$ to the coil to produce a stable time-varying magnetic field. In RX mode the INA163 is connected to the coil and is used to amplify the induced voltage signal to produce $v_{s}\left(t\right)$. (**b**) Layout of the switching circuit on the PCB.

The ADG1436 analog switch (Analog Devices, Norwood, MA, USA) was used to enable the switching functionality. This switch was chosen due to its small PCB footprint, high current carrying capacity and low $R_{on}$ resistance. The switch is controlled using a logic signal controlled by a microcontroller. In TX mode the switch connects the coil to the driver circuit of the tracking system. In RX mode the switch routes the induced coil signal to an instrumentation amplifier for conditioning and amplification. The INA163 (Texas Instruments, Dallas, TX, USA) was selected for amplification due to its high input impedance and low noise characteristics. The gain of the amplifier was set to 81 using a resistor with 1% tolerance, resulting in a maximum gain deviation of ±1.6 using the amplifiers gain Equation (13). A simplified circuit diagram of the switching circuit can be found in Figure 7a. The switch implementation for a single transmitter coil is shown in Figure 7b.

(13) $$G=1+\frac{6000}{R_{G}}$$

### 4.2. Distorter Selection

**Figure 8:**
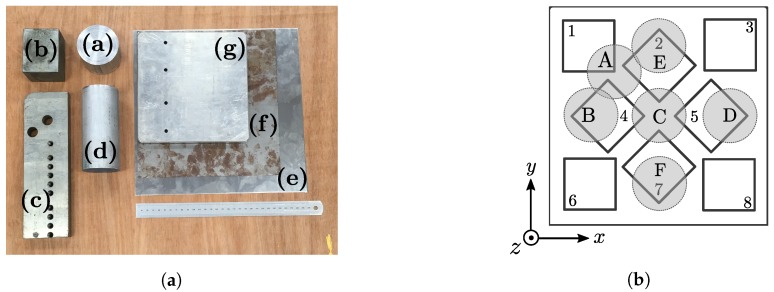
(**a**) Photograph of the metallic distorters used in the experiment. The properties of each distorter are shown in Table 2. (**b**) Illustrated top-down view of the transmitter board showing the locations of the distorters used in experiments.

**Figure 9:**
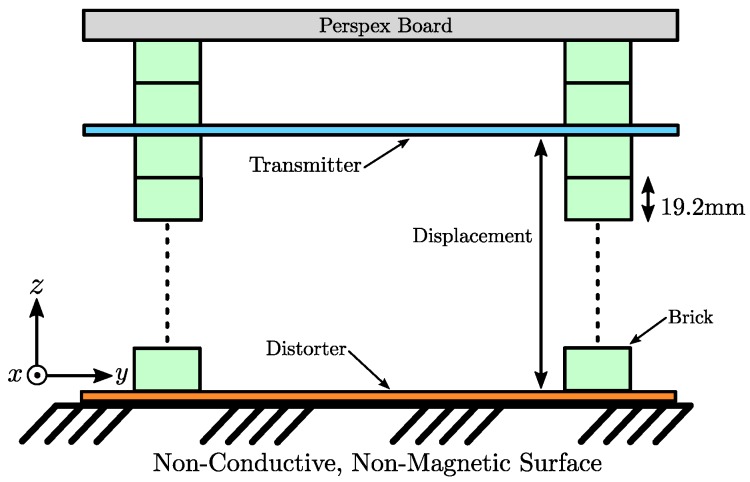
Side-view diagram of the modified transmitter board. The Duplo bricks allow adjustment of the displacement between the transmitter and distorter in increments of 19.2 mm. The Perspex board height is also adjustable, although this was kept at a constant height during the position error experiments. The experimental setup was placed on an all-wood table.

A broad selection of metallic objects were chosen in order to investigate the effects of different materials on the mutual inductance of the transmitter. Both ferrous and non-ferrous metal objects of various dimensions were chosen in order to simulate different scenarios in which distortion error may occur. A photograph of the tested distorters is shown in Figure 8a with the details of each distorter found in Table 2. Each object was fixed in a known position beneath the transmitter board for each measurement. The distance between the distorter under test and transmitter board was varied in fixed *z*-axis increments using Duplo bricks (The Lego Group, Billund, Denmark), with each brick measuring 19.2 mm in height as shown in Figure 9. The tight 10 μm manufacturing tolerance of Duplo [28] ensures repeatability of the experiments while allowing accurate adjustment of the transmitter height displacement. Locations of the distorters with respect to the transmitter *x*-*y* plane can be seen in Figure 8b. Large distorters span the length of the transmitter board and therefore lie underneath more than one labelled location. For example, the steel slab (c) spans lengthways from locations B to D while the sheet distorters span the entire area of the transmitter.

### 4.3. Acquisition Hardware

**Figure 10:**
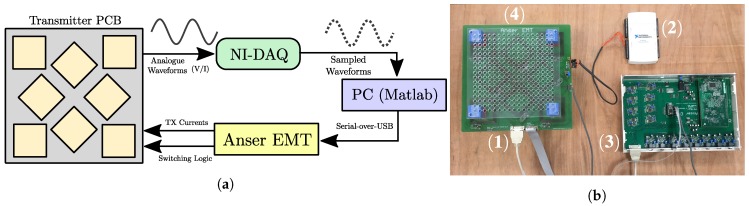
(**a**) Block diagram of the experimental setup. (**b**) Photograph of the experimental setup of the modified transmitter board connected to the Anser EMT system. (1) The modified transmitter board with extra connections to enable coil switching. (2) The National Instruments DAQ connected to the instrumentation amplifier outputs of the transmitter. (3) The Anser EMT tracking system unit with embedded Teensy 3.2 controller. (4) 6 mm Perspex planar board used for sensor position acquisitions.

A photograph of the experimental setup is shown in Figure 10. An Anser EMT system unit was used to drive a constant amplitude alternating current through the transmitting coils. The embedded Teensy 3.2 microcontroller (PJRC, Sherwood, OR, USA) was used to automate the switching the coils between TX and RX modes for the acquisition process. A National Instruments USB-6216 data acquisition unit was used to record the $v_{s}\left(t\right)$ signal of each RX coil, while simultaneously monitoring the TX coil current. The current signal was set to a frequency of 20 kHz for all measurements. The DAQ was configured with a sampling rate of 50 kHz. 5000 samples were used for each individual signal measurement. A minimum signal-to-noise ratio (SNR) of 80dB was observed in the worst case where the furthest separated coil pairs (e.g., Coils 1 and 8) are transmitting and receiving respectively.

## 5. Acquisition Methods

### 5.1. Mutual Inductance Measurement

Switching the EMT system transmitting coils between TX and RX modes was performed iteratively in order to measure the induced voltages on each coil with respect to every other coil. The algorithm as outlined in Algorithm 1 was applied to each distorter location in Table 2 at differing heights in multiples of 19.2 mm as shown in Figure 9. The minimum heights in each case were limited by the size and shape of each distorter, with the lowest minimum height of one Duplo brick (19.2 mm) applied only to the sheet distorters (e)–(g). Distorters (a)–(d) required a minimum of three bricks of separation to avoid touching the transmitter. The output of the algorithm is a matrix of voltage amplitudes and phases corresponding to a particular distorter location and height. The phase information indicates the angle between the transmitter (primary) coil current and induced (secondary) voltage waveforms. The algorithm was performed using Matlab and the Data Acquisition Toolbox. Total acquisition time was approximately 10 s for each distorter configuration. The mutual inductance matrix for the transmitter can then be calculated from the experimentally recorded voltage matrix V using Equation (7).

**Algorithm 1:** Coil switching and acquisition procedure. Acquisition is skipped if indices α=β since this matrix entry represents a coil’s self-inductance.

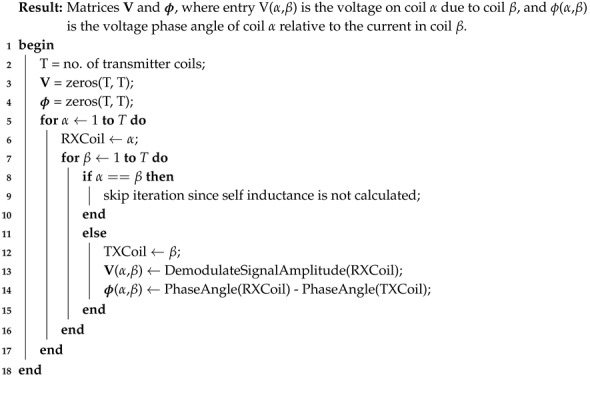



### 5.2. Position Acquisition

Position measurements were also acquired in the presence of each distorter in order to correlate the inductive and phase measurements with the position error experienced by the tracking system. This test requires that all transmitter coils are operating in TX mode with each transmitter coil emitting a unique frequency between 20 kHz and 30 kHz [17,18]. The planar Perspex board shown in Figure 10 was set at a fixed height above the transmitter. The positions were acquired by moving a commercial magnetic sensor (Model No. 610099, Northern Digital Inc., Waterloo, ON, Canada) in a ‘scribbling’ motion on the perspex board to acquire a planar array of *x*-*y* data points at a fixed *z*-displacement above the transmitting field generator. This was repeated for each distorter at the various distorter positions described in Table 2. 1000 random position coordinates were recorded for each measurement. A control acquisition with no distorter present was also acquired.

## 6. Results

### 6.1. Mutual Inductance Matrix

**Figure 11:**
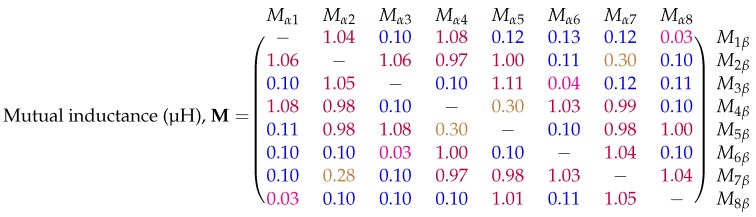
Experimentally calculated mutual inductance matrix for the eight coil magnetic transmitter of the Anser EMT system. The entries have units of microhenries (μH). The diagonal entries are left blank as the system is not built for calculating self-inductances. Results have been truncated and colour-coded for clarity.

The mutual inductance matrix calculation was carried with no distorter present in order to verify the results of the simulation provided by Figure 5. The experimental mutual inductance matrix was calculated using the measured voltages in Equation (7) with the results shown in Figure 11. The symmetry of this matrix matches closely with that of the simulated results. Four unique mutual inductance values can also be identified using the colour-coding scheme used previously.

The simulated and experimental results compare favourably, with corresponding matrix entries experiencing very similar inductance values. The absolute accuracy of the measurement is not critical due to the nature of the inductance error calculation, as only changes in inductance are considered when calculating the error.

### 6.2. Inductance Error

The presence of a conductive or magnetic distorter near the system transmitter board will cause the mutual inductance between the coils to deviate from the control measurement. The magnitude of the change is related to the severity of the distortion. This deviation from the control can be captured by calculating the mutual inductance error matrix **M_e_** in Equation (14).

**M_e_** = **M_distort_** − **M_control_** (14)

where **M_distort_** is the measured mutual inductance matrix with a metallic distorter present and **M_control_** is the measured inductance matrix with no distorter present. Normalising this error with respect to the control measurement in Equation (15) gives the element-wise percentage mutual inductance error with respect to the control.

**M_e%_** = (**M_e_**/**M_control_**) × 100% (15)

The resulting matrix allocates a percentage inductance error to each mutual inductance measurement. The mutual inductance mean error (ME) per coil can be calculated by taking the column summation of **M_e%_** in Equation (16). The ME was used as opposed to the mean absolute error (MAE) so that net average increases and decreases in mutual inductance can be distinguished. Calculating the row summation is also correct since inductance matrices are symmetric by nature. The resulting ME vector allocates each coil a signed mean percentage inductance error.

(16) $$Mean\;Error\%=[\begin{array}{c} \sum_{\beta =1}^{8}M_{{e\%}_{1\beta }}\\ \vdots \\ \sum_{\beta =1}^{8}M_{{e\%}_{8\beta }} \end{array}]/8$$

**Figure 12:**
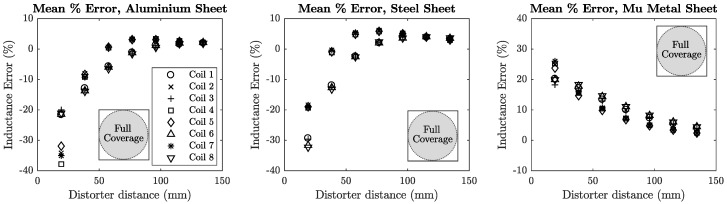
Mutual inductance error for planar sheet distorters. The Mu-metal sheet is the only material to consistently increase the mutual inductance of the transmitter coils.

**Figure 13:**
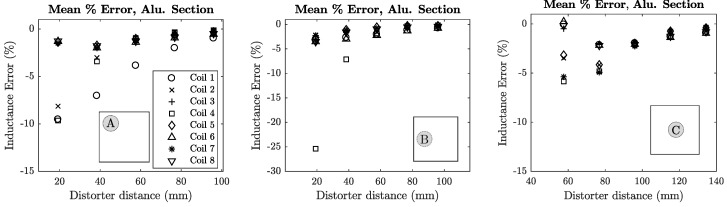
Mutual inductance error for the small aluminium block distorter in locations A, B and C. It can be seen that the location of the distorter decreases the mutual inductance of the coils in closest proximity.

The inductance error of each coil was plotted against the distance of the distorter from the transmitter for each distorter location. These locations are denoted in each plot using Figure 8b as a reference. Mean error plots for sheet metal and aluminium section distorters are shown in Figure 12 and Figure 13.

**Figure A1:**
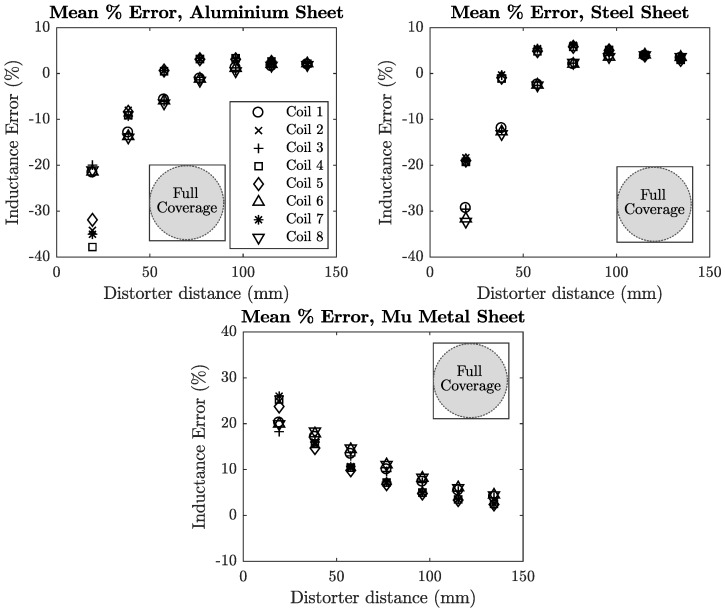
Mutual inductance error plots for planar sheet distorters spanning the area of all transmitter coils. The mu-metal sheet contributes positively to the overall inductance of the transmitter over all distances.

**Figure A2:**
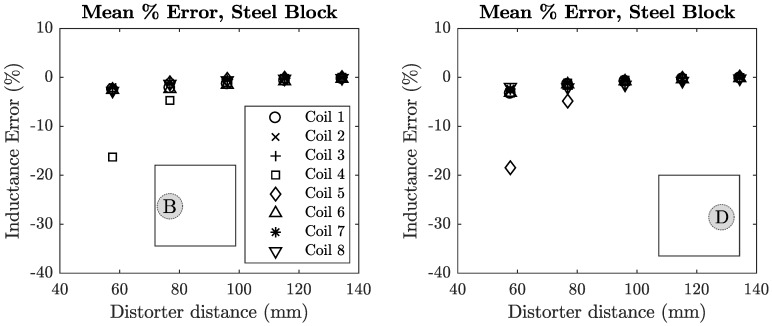
Mutual inductance error plots for small steel block distorter in locations B and D, with the corresponding coils 4 and 5 respectively experiencing the largest mutual inductance error.

**Figure A3:**
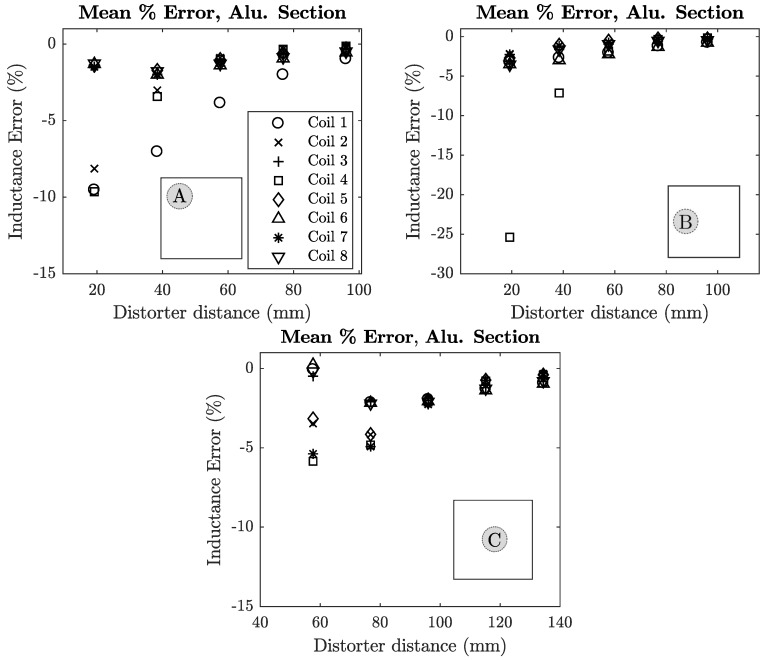
Mutual inductance error for small aluminium block distorter in locations A, B and C. The distorter placed at locations A and B experience the largest errors in their nearest respective coils, 1 and 4. The centrally placed distorter at location C causes the central transmitter coils 2, 4, 5 and 7 to experience the largest mutual inductance error.

**Figure A4:**
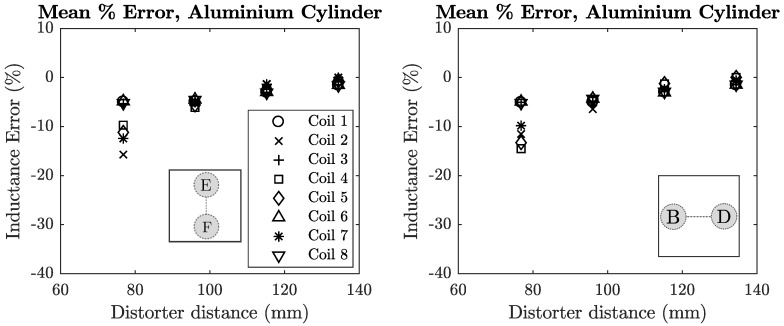
Mutual inductance error plots for large aluminium cylinder in locations E to F and B to D. The cylinder spans the length of the transmitter board. The highest mutual inductance errors correspond to the positions of the distorter.

**Figure A5:**
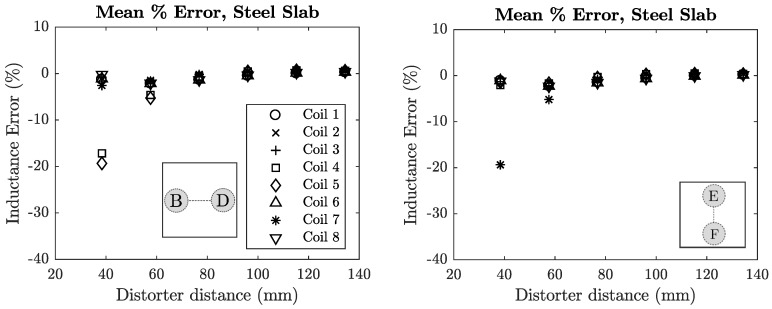
Mutual inductance error plots for steel slab in locations B to D and E to F. The effect is similar to that of the aluminium cylinder, with the highest error experienced by the coils in closest proximity to the distorter.

Results for the other remaining distorters can be found in Figure A1, Figure A2, Figure A3, Figure A4 and Figure A5 in Appendix A.

### 6.3. Phase Response

The demodulation process used to acquire the induced voltage signal magnitudes also yields the phase angle between the transmitter coil current and the corresponding mutually induced voltage waveforms. The phase angle *θ* between these current and voltage waveforms is ideally *π*/2 radians in the absence of distorters. This phase angle, similar to the inductance, will deviate depending on the magnitude of the distortion and the material of the distorting object. Subtracting the phase angle of the control measurement from the distortion measurement in Equation (17) yields a matrix of phase differences for each mutual inductance entry.

***ϕ_e_*** = ***θ_distort_*** − ***θ_control_*** (17)

where ***θ_distort_*** is a matrix of calculated phase angles between the driving coil current and mutually induced voltages when a distorter is present and ***θ_control_*** are the corresponding phase angles with no distorter present. The mean coil phase error ***ϕ_e_*** is then calculated in Equation (18).

(18) $$Mean\;Phase\;Error\;({}^{\circ})=[\begin{array}{c} \sum_{j=1}^{8}\phi_{e_{1j}}\\ \vdots \\ \sum_{j=1}^{8}\phi_{e_{8j}} \end{array}]/8$$

**Figure 14:**
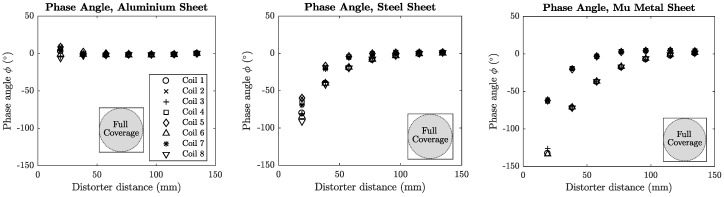
Phase error for the planar sheet distorters. The aluminium sheet contributes a small positive phase shift, while both steel and mu-metal sheets contribute large negative phase shifts when in close proximity to the transmitter.

**Figure 15:**
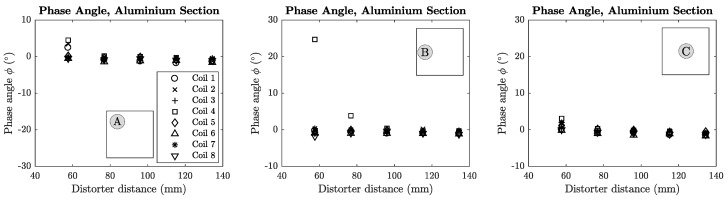
Phase difference plots for small aluminium block distorter in locations A, B and C.

The phase error of each coil was plotted against the distance of the distorter from the transmitter for each distorter location. These locations are denoted in each plot using Figure 8b as a reference. Mean phase error plots for sheet metal and aluminium section distorters are shown in Figure 14 and Figure 15.

**Figure A6:**
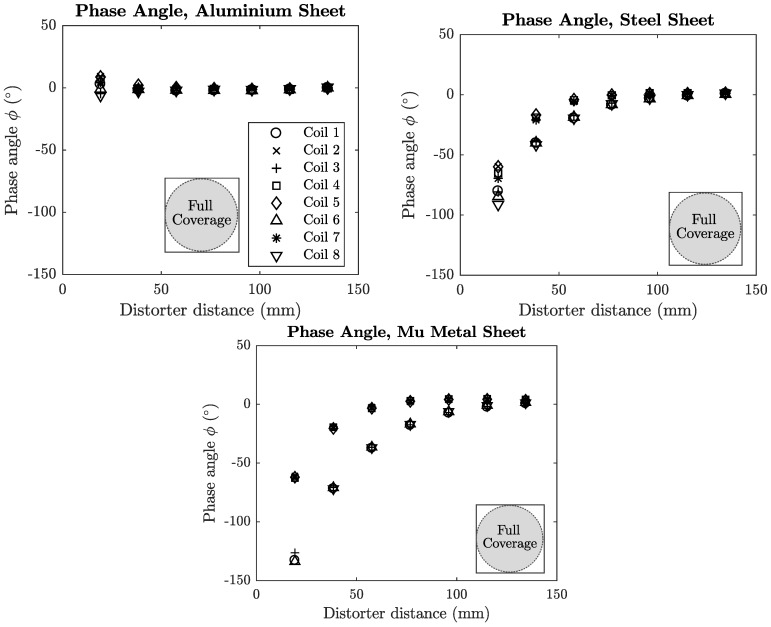
Phase error for planar sheet distorters. The aluminium sheet causes a small positive phase error of approximately 10${}^{\circ }$ when placed very close to the transmitter, but quickly returns to zero with increasing distance. The steel sheet initially causes very large negative phase errors when close to the transmitter but return to zero at approximately 120 mm from the transmitter. The mu-metal sheet phase error is an exaggerated version of that of the steel sheet, with maximum errors of approximately 140${}^{\circ }$.

**Figure A7:**
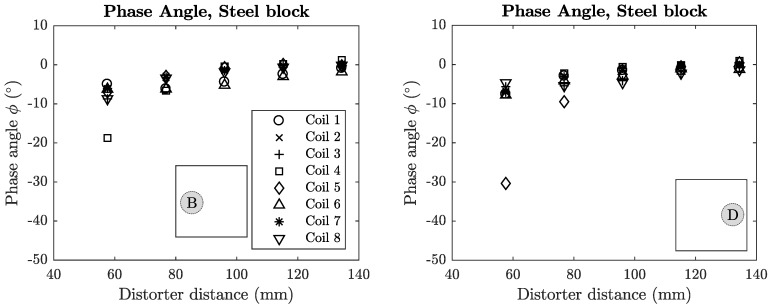
Phase error plots for small steel block distorter in locations B and D. The largest errors are experienced by coils 4 and 5 in closest proximity to locations B and D respectively. Negative phase error is experienced in both locations.

**Figure A8:**
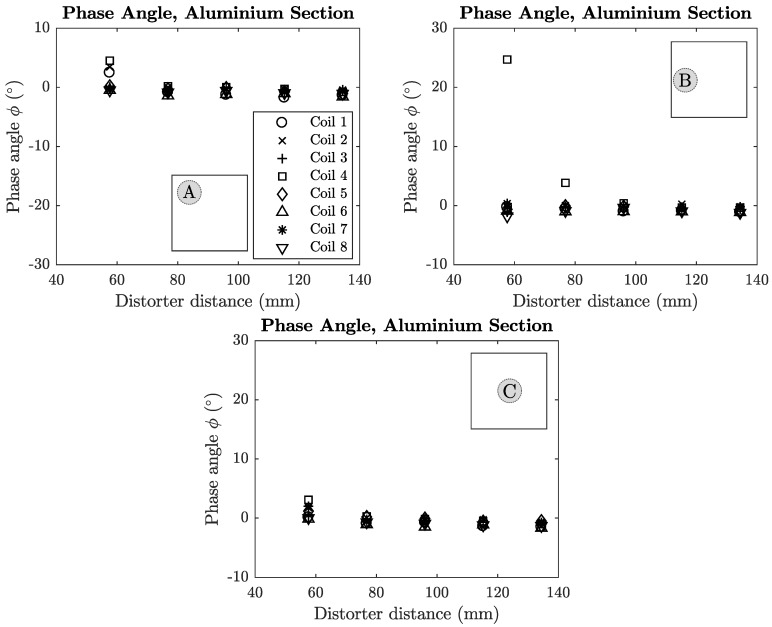
Phase error plots for small aluminium block distorter in locations A, B and C. The largest phase errors are experienced by the in closest proximity to locations A, B and C. Positive phase error is experienced in all cases.

**Figure A9:**
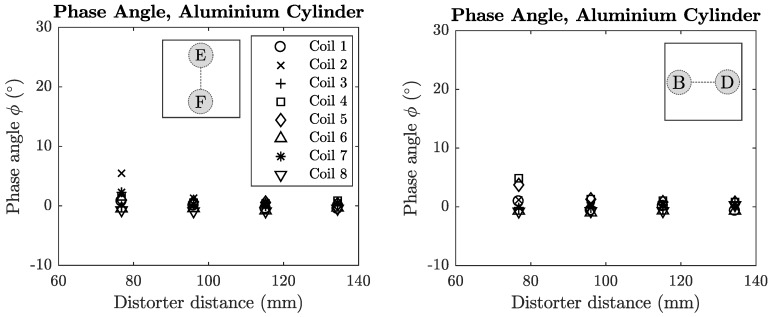
Phase error plots for large aluminium cylinder in locations E to F and B to D. The cylinder spans the length of the transmitter board. The highest phase errors correspond to the positions of the distorter. Positive phase error is experienced in each case.

**Figure A10:**
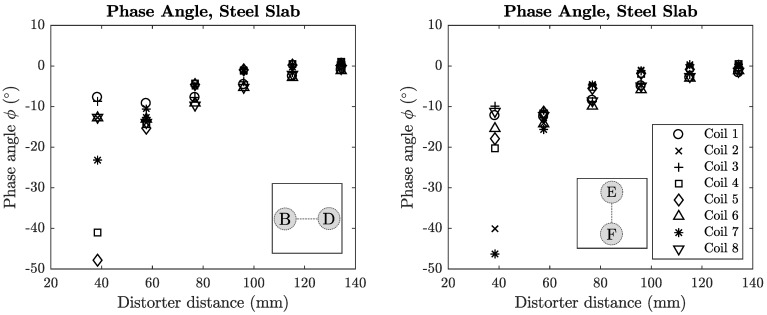
Phase error plots for steel slab in locations B to D and E to F. Negative phase is experienced in each case, with the highest phase error experienced by the coils in closest proximity to the distorter.

The phase plots for the remaining distorters can be found in Figure A6, Figure A7, Figure A8, Figure A9 and Figure A10 in Appendix B.

### 6.4. Position Error

The planar position acquisitions provided point clouds in the *x*-*y* plane of the transmitter at a fixed *z*-displacement. For a given distorter configuration, the mean *z*-axis error *e_z_* with respect to the control was calculated by subtracting the mean *z* value of the control, ${\bar{z}}_{control}$, from the distorted cloud positions *z_i_* and averaging over *N* points in Equation (19). The absolute error was not taken in order to preserve the sign of the error. The standard deviation of each distorted point cloud was calculated with respect to its own mean ${\bar{z}}_{distort}$ using Equation (20).

(19) $$e_{z}=\frac{1}{N}\sum_{i=0}^{N}(z_{i}-{\bar{z}}_{control})$$

(20) $$\sigma_{z}=\frac{1}{N}\sqrt{\sum_{i=0}^{N}{\left(z_{i}-{\bar{z}}_{distort}\right)}^{2}}$$

**Figure 16:**
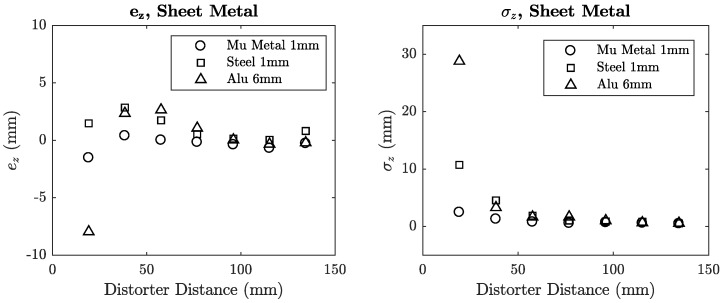
Mean error and standard deviation for sheet metal distorters.

**Figure 17:**
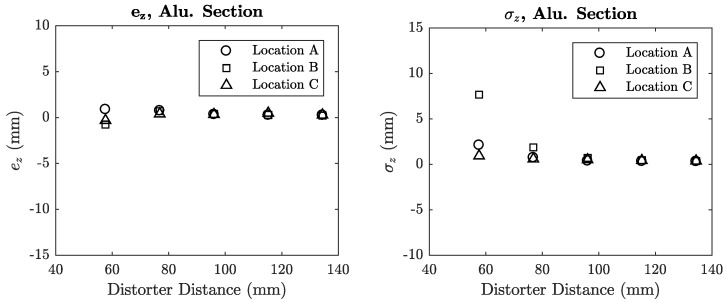
Mean error and standard deviation for aluminium section distorter in locations A, B and C.

**Figure 18:**
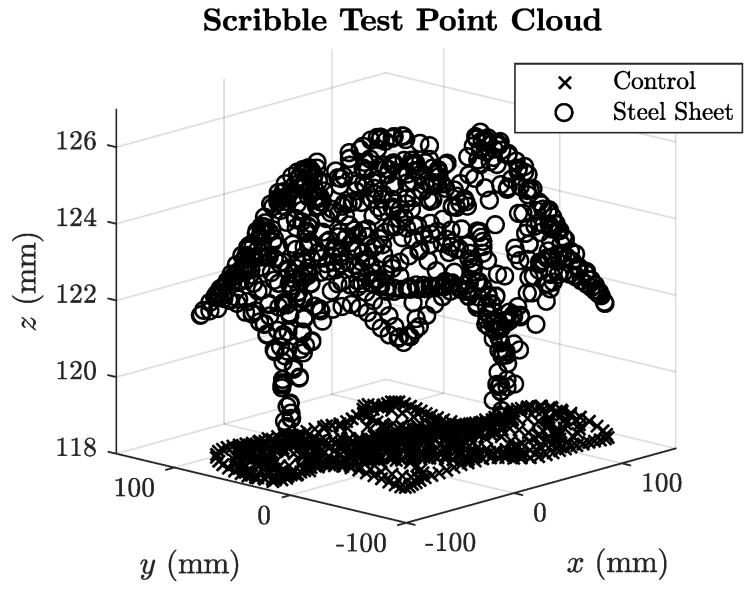
Point clouds a scribble test position acquisition for the 1 mm steel sheet placed 57 mm below the transmitter PCB. The point cloud with steel present has a mean height of ${\bar{z}}_{steel}$ of 129.5 mm and large standard deviation of $\sigma_{z_{steel}}=7.46$ mm. The control point cloud is also shown for reference (${\bar{z}}_{control}=118.5$ mm, $\sigma_{z_{control}}=0.37$ mm).

The plots for both sheet and aluminium distorters are shown in Figure 16 and Figure 17. High values of *σ_z_* indicate large variances in the error when the distorter is near the transmitter board. This error variance is visualised in the example point cloud plot in Figure 18 with and without the 1 mm steel sheet, where its presence causes a large deviations in position when compared to the control where no distorter is present.

**Figure A11:**
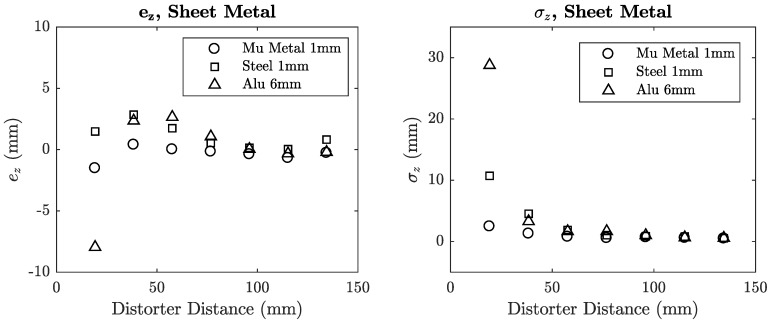
Position error plots for sheet metal distorters. Aluminium causes the most severe position error and highest standard deviation. A cross-over point is visible at approximately 100 mm in which the errors are minimised, before increasing for distances >100 mm.

**Figure A12:**
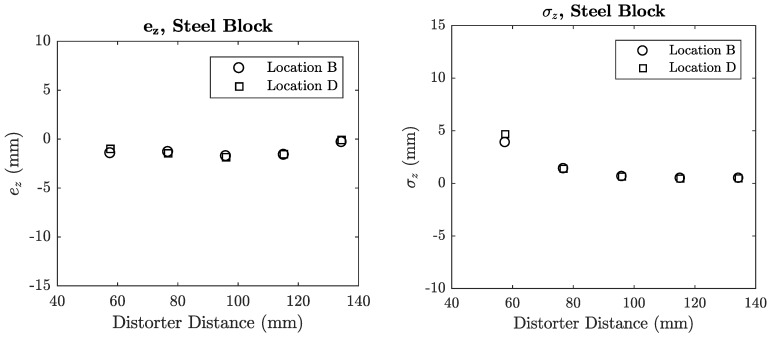
Position error plots for steel block, locations B and D.

**Figure A13:**
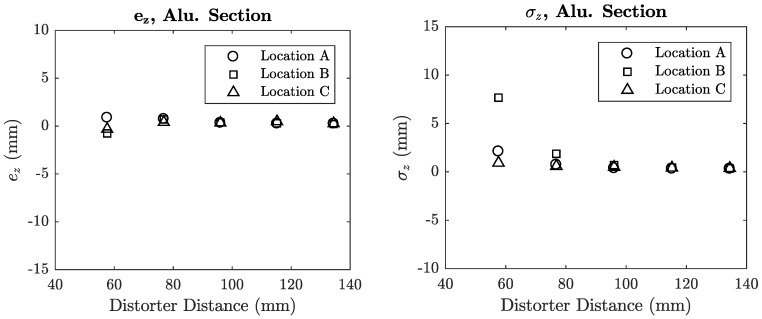
Position error plots for aluminium section in locations A, B and C. Location B causes the highest positioning error and standard deviation as the aluminium section is placed directly below transmitter coil 4.

**Figure A14:**
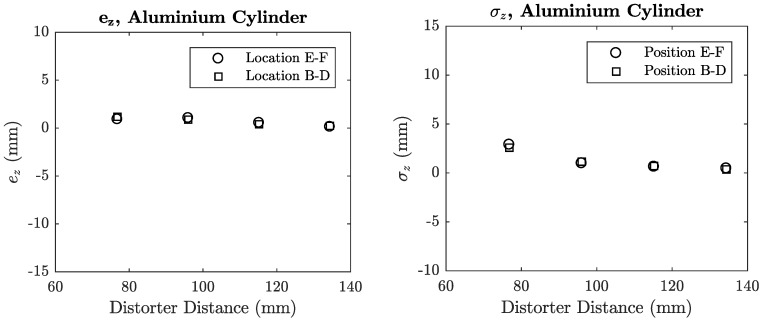
Position error plots for aluminium, cylinder, locations B and D and E to F.

**Figure A15:**
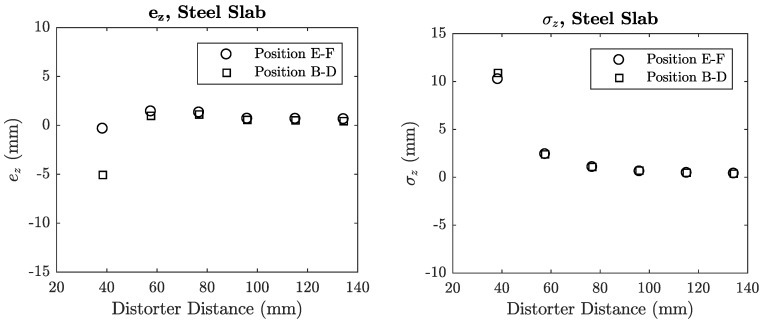
Position error plots for steel slab, locations B and D and E to F.

Position error plots for the remaining tested distorters can be found in Figure A11, Figure A12, Figure A13, Figure A14 and Figure A15 in Appendix C.

## 7. Discussion

Comparing the data of both inductance matrix and position experiments highlights a number of useful relationships between the properties of the distorters and the transmitter coils.

### 7.1. Error vs. Distance

There is an excellent correspondence between the measured inductive and positional error in relation to the distorter’s distance from the transmitter. In all cases it can be clearly seen that placing a distorter nearer to the transmitter will increase both the inductance and positional errors. This is intuitively correct as a distorter’s interaction with the transmitter’s magnetic field will become increasingly significant the closer it is placed to the field source. Steel and aluminium distorters tend to give rise to negative *z*-position error, in which the distorter negatively impacts the mutual inductance of the transmitter, although it can be seen in Figure 12 that a slight positive inductance contribution is evident for *z*-positions greater than 50 mm. The mu-metal sheet contributes positively to the transmitter’s mutual inductance for all *z*-positions. These results strongly agree with [12] in which the SNR of the tracking system sensor is increased when a mu-metal shield is used.

### 7.2. Phase Angle vs. Material

A significant relationship can be found between the experimentally calculated phase angle and the material of the distorter. All steel distorters exhibited negative phase angles in these experiments, while the aluminium distorters experienced all positive phase angles. The mu-metal sheet distorter introduced very significant negative phase compared to the steel sheet, with a total phase angle of approximately $-140^{\circ }$. Clearly the phase angle and permeability of the material have a positive correlation and can be used to determine whether the distorter is ferromagnetic or purely conductive in nature. These results compare favourably with those of [24] which showed that purely conductive materials introduce opposing phase to that of ferrous materials.

### 7.3. Inductance & Phase Error vs. Distorter Location

A strong correlation was shown between the location of a distorter and the error that it produces. This is particularly relevant to the small steel and aluminium distorters. In the case of the aluminium section and steel block distorters, the transmitter coils that are in closest proximity experience the highest inductance (and phase) errors. This holds for the larger steel slab and aluminium cylinder distorters, although the relationship becomes less significant with increasing distance from the transmitter. In particular, Figure 13 shows that the highest inductance errors for a distorter in position A are experienced by coils 1, 2 and 4 which are directly above the distorter as shown in Figure 8b. From these observations we can say that there is a correlation between the location of the distorter and the location of the coil(s) experiencing the highest inductance error. This relationship may be useful when initially placing the system transmitter in order to minimise position errors from local distorters. Further to this, the relationship may also enable EMT users to create quantitative measures of tracking confidence in the presence of distorters based on their location, in which specific portions or sub-volumes of the full EMT tracking volume are assigned a confidence score derived from the nearest transmitter coil’s inductance error. It should be noted that the analysis presented in this paper is most effective when distortions are of a static nature, as the method assumes the environment does not change during the inductance measurement procedure.

## 8. Conclusions

This paper presented a novel electromagnetic tracking system transmitter capable of characterising the nature of distortions in close proximity without the use of a standalone magnetic sensor. A mutual inductance model for the system transmitter was developed, followed by an experimental amplitude and phase analysis of the system transmitter coils. The results allow the user to distinguish the size, severity and material (ferromagnetic or non-ferrous conductor) of the distorting objects in close proximity to the system transmitter. The presented analyses can be readily applied to other electromagnetic tracking transmitter designs if induced voltages can be measured on all constituent transmitter coils. Achieving material discrimination in this manner may allow researchers to craft error compensation methods which are specifically designed for a particular distorter configuration. Future work will see the these analyses integrated in a software toolkit for the Anser EMT system, allowing optimal placement of transmitters to minimise the effect of environmental distorters in the clinical setting.

## Figures and Tables

**Table 1:** Properties of EMT transmitter coils.

Property	Value	Unit
Length (Max.)	70	mm
Width (Max.)	70	mm
Turns	25	-
Inductance	70	μH
Resistance (coil)	1.5	Ω
Resistance (switch)	3	Ω
Resistance (Total)	4.5	Ω
Trace Width	1.6	mm
Trace Spacing	0.25	mm

**Table 2:** List of distorters used in the experiment.

Label	Distorter Type	Name	Material	L (mm)	W (mm)	H (mm)	Locations
(a)	Small	Alu. Section	Aluminium	70	70	48	A, B, C
(b)	Small	Steel Block	Mild Steel	60	70	50	B, D
(c)	Medium	Steel Slab	Mild Steel	250	75	23	B-to-D, E-to-F
(d)	Medium	Alu. Cylinder	Aluminium	140	63	-	B-to-D, E-to-F
(e)	Sheet	Mu Sheet.	Mu Metal	300	300	1	Full coverage
(f)	Sheet	Steel Sheet	Mild Steel	265	250	1	Full coverage
(g)	Sheet	Alu. Sheet	Aluminium	195	195	6	Full coverage

## Appendix A. Inductance Error Plots

The inductance error plots for the sheet and aluminium section distorters were shown in the main article. These plots are repeated here with the remaining mutual inductance error plots for the distorters in Table 2. In nearly all cases the mean mutual inductance error calculated using Equation (16) decreases with decreasing distance between the distorter and transmitter. It can also be seen that the smaller distorters cause the highest percentage error in the transmitter coils that are in closest proximity.

## Appendix B. Phase Angle Plots

The phase angle error plots for the sheet and aluminium section distorters were shown in the main article. These plots are repeated here with the remaining phase error plots for the distorters in Table 2 calculated using Equations (17) and (18). The resulting angle allows for a clear differentiation between materials. Aluminium distorters cause a positive phase error in all cases, while steel distorters cause negative phase error.

## Appendix C. Position Error

The position error plots for the sheet and aluminium section distorters were shown in the main article. These plots are repeated here with the remaining position error plots for the distorters in Table 2. Equations (19) and (20) were used to extract the mean error $e_{z}$ and standard deviation $\sigma_{z}$ from each planar point cloud. The resulting errors show how the different distorters effect the tracking performance of the Anser EMT system.
